# A case of acute myocardial infarction due to left main coronary artery occlusion: Case report

**DOI:** 10.1097/MD.0000000000048353

**Published:** 2026-04-17

**Authors:** Guochun Hu, Di Wang, Taiyun Chu, Yuanyuan Zhou, Yudi Wang, Shihong Luo

**Affiliations:** a Sixth Affiliated Hospital of Kunming Medical University, Yuxi, Yunnan, China.

**Keywords:** acute myocardial infarction, cardiogenic shock, case report, left main coronary artery

## Abstract

**Rationale:**

Left main coronary artery (LMCA)-related acute myocardial infarction with cardiogenic shock (CS) carries >80% mortality without immediate revascularization.

**Patient concerns:**

Thrombolysis often fails in such cases with LMCA and CS, necessitating salvage percutaneous coronary intervention, which typically requires stenting and mechanical circulatory support.

**Diagnoses:**

LMCA and CS.

**Interventions:**

We report a unique case successfully managed with drug-coated balloon (DCB)-only angioplasty amid resource constraints during the pandemic. This report presents a high-risk case of LMCA acute myocardial infarction with CS following unsuccessful TNK thrombolysis at the local hospital, successfully revascularized with DCB-only angioplasty without stenting, intravascular ultrasound (IVUS), intra-aortic balloon pump, or extracorporeal membrane oxygenation, because of pandemic-related medical resource limitations (IVUS/intra-aortic balloon pump unavailable) and extracorporeal membrane oxygenation not yet implemented at our hospital at that time.

**Outcomes:**

Post-procedural TIMI 3 flow was achieved immediately. Postoperatively, the patient’s CS gradually resolved. After 1 month, IVUS performed at a tertiary hospital confirmed no need for further intervention for LMCA. Six months later, cardiac function had recovered to normal.

**Lessons:**

This case demonstrates that in resource-limited settings, when safe stent implantation cannot be guaranteed for thrombotic left main occlusion, the DCB-assisted rapid reperfusion strategy may represent a viable, life-saving option. It offers a new approach for primary care hospitals managing such critical emergencies.

## 1. Introduction

Left main coronary artery (LMCA)-related acute myocardial infarction (AMI) complicated by cardiogenic shock (CS) carries an in-hospital mortality exceeding 80% without timely revascularization. Current guidelines universally recommend primary percutaneous coronary intervention (PCI) with stent implantation or coronary artery bypass grafting as the gold standard. In patients with CS complicating AMI due to LMCA, ideally, PCI should be combined with mechanical circulatory support (MCS) devices such as intra-aortic balloon pump (IABP) or extracorporeal membrane oxygenation (ECMO) to stabilize hemodynamics. Adjunctive techniques including thrombus aspiration and intravascular ultrasound (IVUS)-guided optimization are frequently employed to improve outcomes. It is important to stress how crucial imaging guidance typically is when performing complex LMCA interventions. Intravascular imaging (e.g., IVUS or optical coherence tomography, OCT) is well-documented to improve procedural outcomes by allowing precise assessment of lesion morphology, vessel sizing, and stent optimization.

Thrombolysis therapy remains a critical bridging intervention in non-PCI-capable centers. However, its efficacy in LMCA-AMI is notably poor (reperfusion success < 40%), and failure often precipitates rapid clinical deterioration.

This report presents a high-risk case of LMCA-AMI with CS following unsuccessful recombinant human TNK tissue-type plasminogen activator (rhTNK-tPA) thrombolysis, successfully revascularized with drug-coated balloon (DCB)-only angioplasty during pandemic-related resource limitations – without stenting, IVUS, IABP, or ECMO – demonstrating a potential rescue strategy in constrained environments.

## 2. Case presentation

A middle-aged male patient complaining of “chest pain for about 2 hours” came to our hospital and was transferred to our department by emergency medical services. He was in his 40s with a 20-pack-year smoking history but denied any history of hypertension, diabetes, or hyperlipidemia.

This patient’s prehospital treatment was as follows.

**Figure 1.**
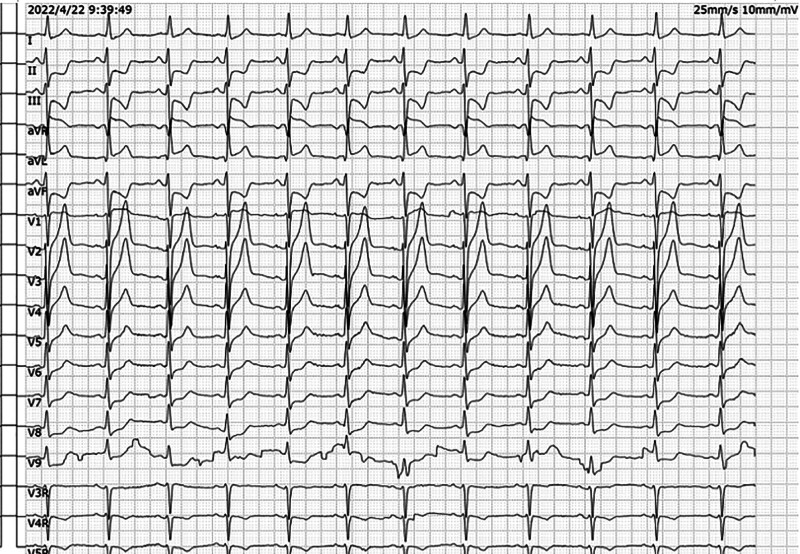
(A) Sinus rhythm. (B) PR-segment elevation in lead aVL and PR-segment depression in leads II, III, and aVF, suggesting atrial infarction. (C) ST-segment elevation in leads aVR and V1. The magnitude of ST-segment elevation in lead aVR was greater than that in lead V1. (D) The horizontal or downward-slanting depression of ST segments in leads II, III, aVF, and V5–V9 was ≥0.05 mV, and ST-segment depression was more pronounced in inferior wall leads. (E) The T waves in the leads V1–V4 were tall, broad and upright. (F) Left anterior branch block.

**Figure 2.**
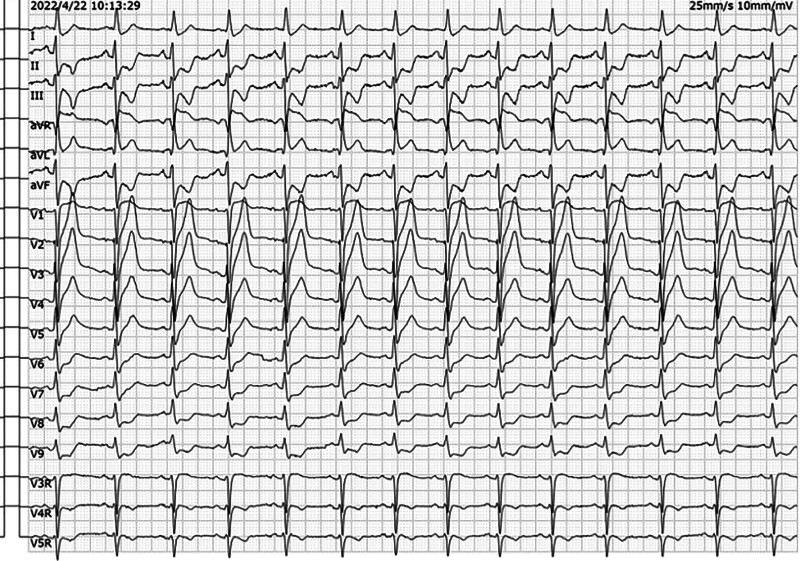
(A) Sinus rhythm. (B) PR-segment elevation in leads aVL and aVR and PR-segment depression in leads II, III, and aVF, suggesting atrial infarction. (C) ST-segment elevation leads to aVR, V1, and V3R. The magnitude of ST-segment elevation in lead aVR was greater than that in lead V1. ST-segment depression ≥0.05 mV in leads II, III, aVF, and V5–V9; horizontal or downward-sloping and more significant in inferior wall leads. (D) The T waves in the V1–V4 leads were tall, broad and upright. (E) Left anterior branch block. (F) In comparison with Figure 1. the ST segments in leads aVR and V1 were more elevated, and those in leads II, III, aVF, and V5–V9 were more depressed.

Electrocardiogram (ECG) at a local hospital revealed LMCA-AMI because of the ST-segment elevation in leads aVR and V1, the magnitude of ST-segment elevation in lead aVR was greater than that in lead V1, the horizontal or downward-slanting depression of ST segments in leads II, III, aVF, and V5-V9 was ≥0.05 mV, and ST-segment depression was more pronounced in inferior wall leads. The T waves in leads V1 to V4 were tall, broad and upright (Fig. 1). Treatments were given at a local hospital. First, 300 mg aspirin tablets, 300 mg clopidogrel tablets and 40 mg atorvastatin tablets were taken orally. Second, 5000 IU normal sodium heparin was pushed intravenously. Third, 16 mg rhTNK-tPA was pushed intravenously for thrombolysis.^[1,2]^ After this series of rescue treatments, the patient’s symptoms, such as chest pain and sweating, were not reduced. In comparison with Figure 1, the ST segments in leads aVR and V1 were more elevated, and those in leads II, III, aVF, and V5 to V9 were more depressed. The T waves in leads V1 to V4 were still tall, broad and upright on repeat ECG (Fig. 2). Our chief resident of our department initially assessed that the patient had acute occlusion of the LMCA, the infarct-related artery was not recanalized, blood flow was not restored, and reperfusion therapy failed after thrombolysis.

**Figure 3.**
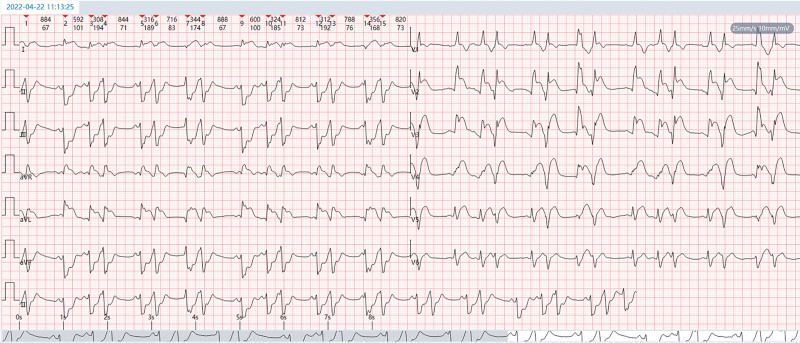
(A) Sinus tachycardia. (B) Frequent premature ventricular beats, mostly appearing bigeminy, occasionally occurring in pairs, with the R-on-T phenomenon. (C) Frequent accelerated ventricular escape beats. (D) Complete right bundle branch block with left anterior branch block. (E) ST-segment elevation in leads aVR, I, aVL, and V1–V6 and horizontal or downward-sloping depression of the ST-segment ≥0.05 mV in leads II, III, and aVF. (F) The ECG characteristics of the hyperacute injury phase of myocardial infarction with extensive anterior wall ST-segment elevation are presented.

**Figure 4.**
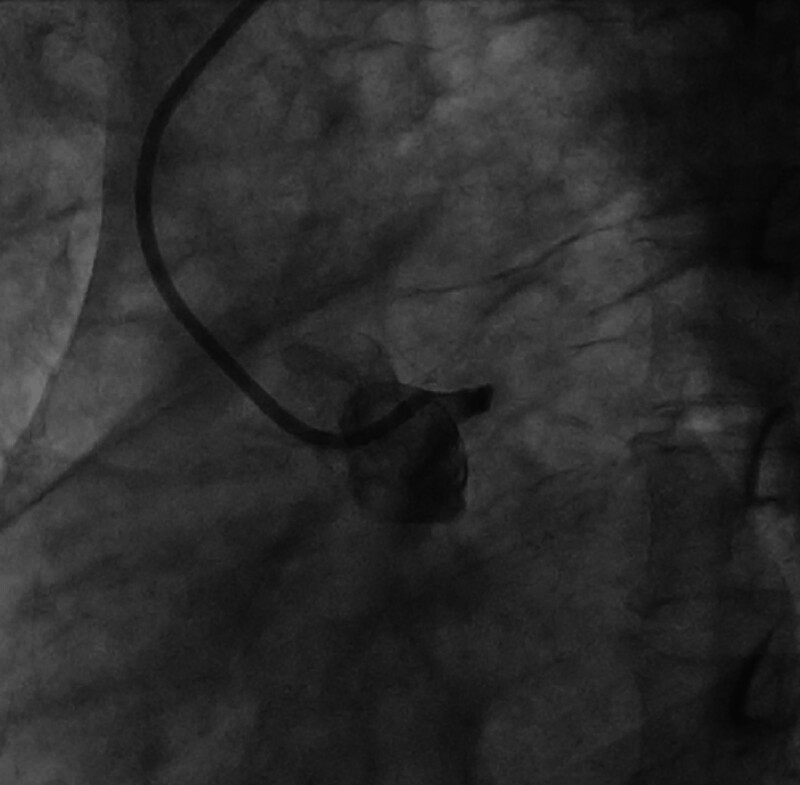
Complete occlusion of the LMCA from the opening. LMCA = left main coronary artery.

**Figure 5.**
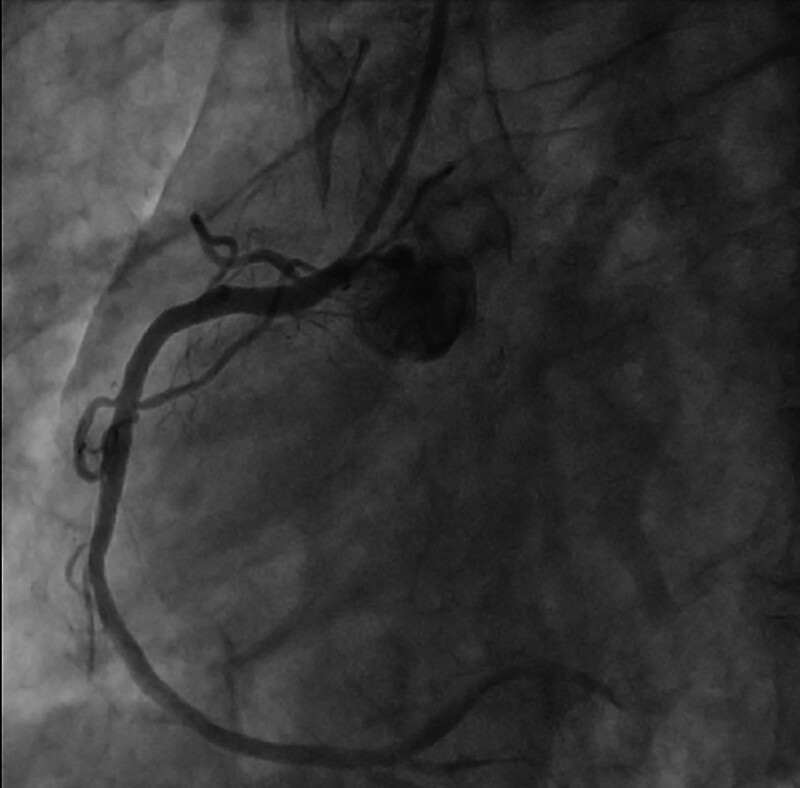
Segmental plaque with 60% stenosis in the middle RCA, with flow TIMI grade 3. RCA = right coronary artery.

**Figure 6.**
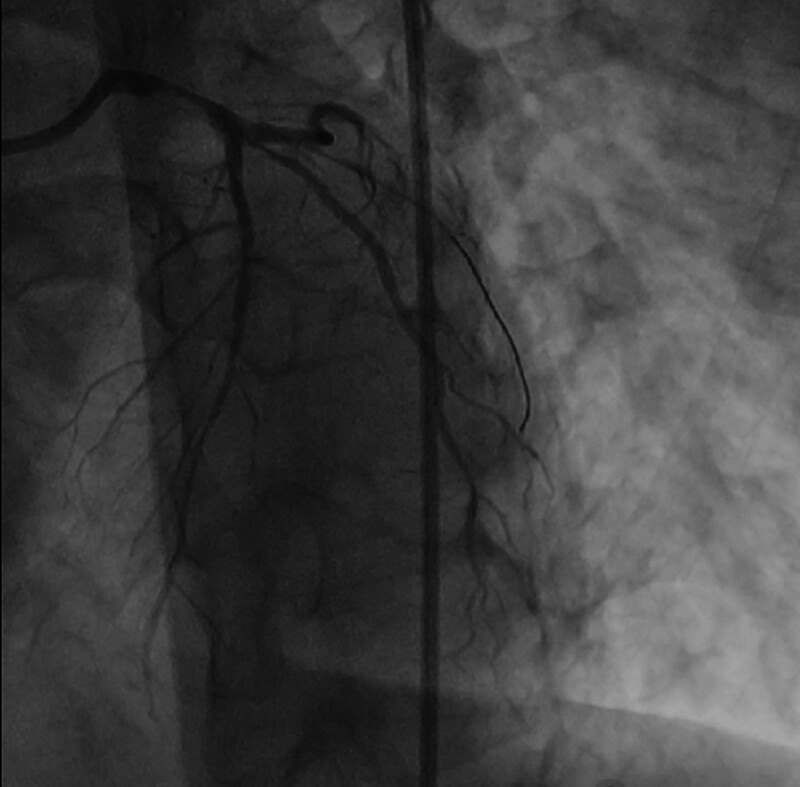
A coronary balloon was delivered to the LMCA lesion, and after predilatation, blood flow was restored to the LMCA and its branches. CAG showed segmental plaque and stenosis of 95% in the proximal-middle portion of the LMCA and stenosis of 90% in the opening of the diagonal branch, with normal blood flow. CAG = coronary arteriography, LMCA = left main coronary artery.

**Figure 7.**
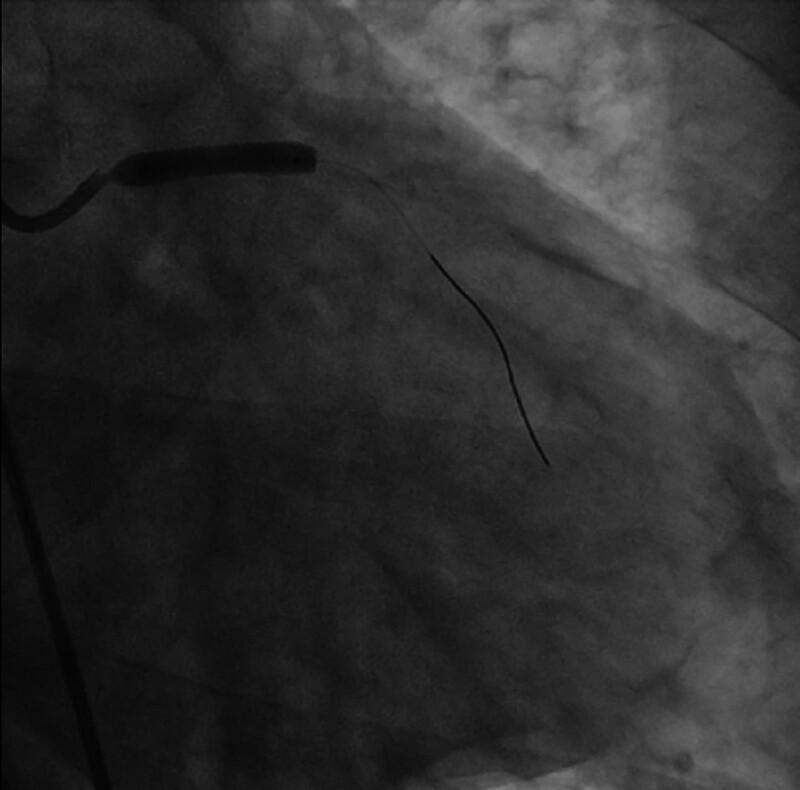
A DCB was selected to be delivered to the patient’s LMCA lesion, and angioplasty was performed. DCB = drug-coated balloon, LMCA = left main coronary artery.

**Figure 8.**
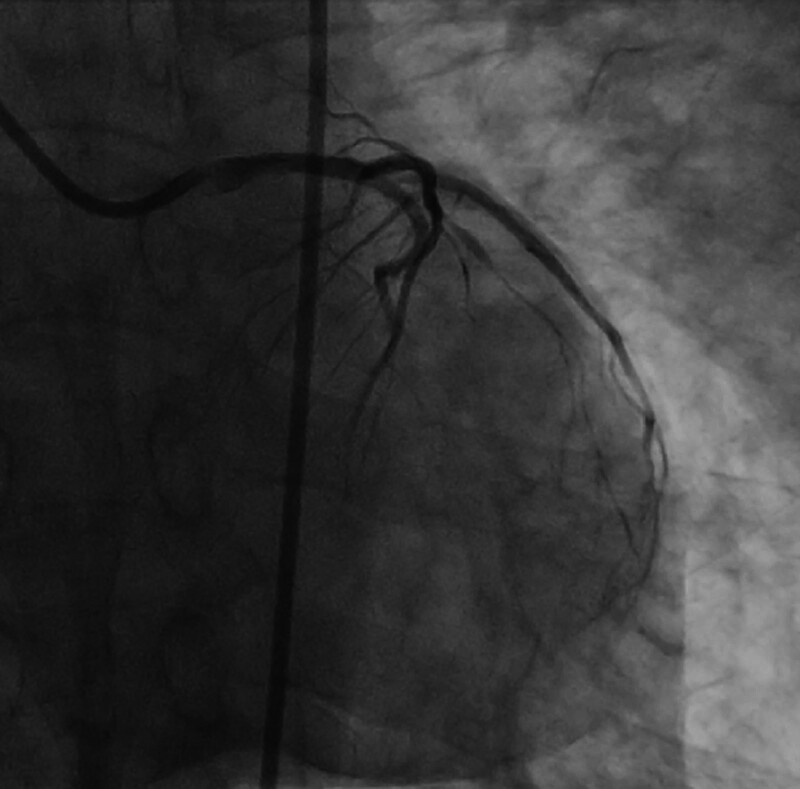
A DCB angioplasty was performed. CAG after the operation was performed. The images showed 30% residual stenosis of the LMCA, with normal blood flow in the LAD, LCX and diagonal branches. CAG = coronary arteriography, DCB = drug-coated balloon, LAD = left anterior descending branch, LCX = left circumflex branch, LMCA = left main coronary artery.

**Figure 9.**
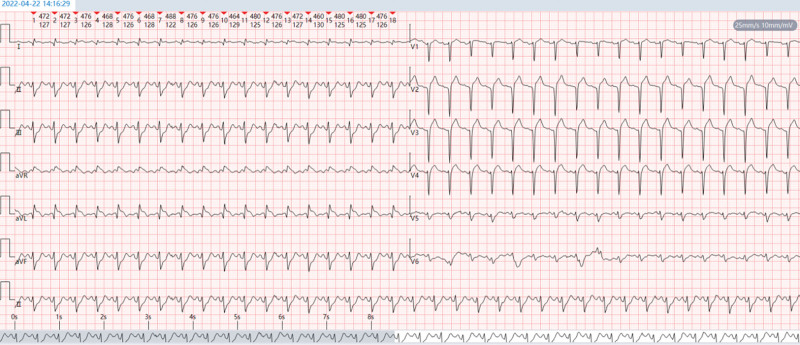
(A) Sinus tachycardia. (B) Acute anteroseptal wall and apical myocardial infarction (QS pattern in leads V1–V3, rS pattern in lead V4, with minimal r-wave time occupancy). (C) Left anterior branch block. (D) Low QRS voltage in leads V5–V6. (E) Compared with Figure 3. the ST-segment regression in leads aVR, I, aVL, and V1–V6, reduced ST-segment depression in leads II, III, and aVF, and ventricular arrhythmia and complete right bundle branch block disappeared.

The emergency team brought the patient straight to the interventional theater, bypassing our emergency department and coronary care unit. Admission physical examination: blood pressure 80/50 mm Hg, oxygen saturation 86%, lethargy, profuse sweating, wet and cold skin, and numerous moist crackles were detected on auscultation over the bilateral lower lung zones. The heart rate was 110 beats per minute (bpm), arrhythmia and frequent premature dystonic beats were heard, heart sounds were low, and no murmurs were heard in the valvular auscultation zones. Repeat ECG: sinus tachycardia; frequent ventricular premature beats, mostly appearing bigeminy, occasionally in pairs; frequent accelerated ventricular escape beats; complete right bundle branch block and left anterior branch block; and extensive anterior ST-segment-elevation myocardial infarction in the supra-acute injury stage (Fig. 3). Admission diagnosis: coronary atherosclerotic heart disease, acute extensive anterior wall myocardial infarction, failure of reperfusion after thrombolysis, combined with CS (case report). The indications for emergency coronary angiography (CAG) and rescue PCI was clear. Immediate emergency CAG via right radial artery puncture showed complete occlusion of the LMCA from the opening (Fig. 4) and segmental plaque and 60% stenosis in the middle of the right coronary artery, with flow TIMI grade 3 (Fig. 5). The members of the PCI team discussed the following: Implanting a stent in this thrombus-laden lesion would significantly increase the risk of early in-stent thrombosis and no-reflow after stenting. Furthermore, forcibly implanting a stent without assessing its fit and size via IVUS would carry a high risk of incomplete stent expansion and poor apposition. Should such adverse events occur, the consequences for the patient would be catastrophic. After predilatation, blood flow was restored to the LMCA and its branches, and it was seen to be 95% stenosed with segmental plaques in the proximal mid-section and 90% stenosed in the opening of the diagonal branch, and its blood flow was normal (Fig. 6). A DCB was delivered to the patient’s LMCA lesion, and angioplasty was performed. Postoperative re-CAG showed 30% residual stenosis of the LMCA, with TIMI 3 blood flow in the left anterior descending branch, left circumflex branch and diagonal branches (Figs. 7 and 8). It took only 15 minutes from the puncture of the right radial artery to successful reperfusion via DCB angioplasty. Postoperatively, the patient’s blood pressure gradually returned, and the CS was gradually corrected. Postoperative repeat ECG: The ST segments of the anterior wall lead were obviously regressed; and The ventricular arrhythmia and complete right bundle branch block disappeared (Fig. 9).

**Figure 10.**
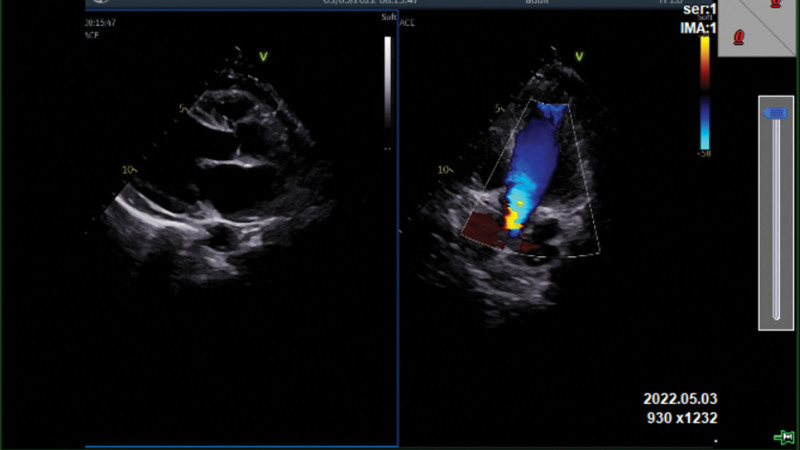
(A) Segmental ventricular wall motion abnormalities. (B) Prethrombosis in the left ventricle. (C) A small amount of pericardial effusion. (D) Reduced left ventricular systolic function: LVEF was 38%. LVEF = left ventricular ejection fraction.

After active rescue treatments, the patient was finally stable. He was discharged with stable vital signs and was in good general condition. At the time of discharge, cardiac ultrasound showed segmental ventricular wall motion abnormalities, prethrombosis in the left ventricle, a small amount of pericardial effusion, and reduced left ventricular systolic function, with a left ventricular ejection fraction of 38% (Fig. 10).

One month later, follow-up CAG at a tertiary hospital confirmed via IVUS that there was no need for further intervention for the LMCA.

We followed up with him by telephone for 6 months. His condition was stable, with no further episodes of chest pain or other symptoms. He was able to take care of himself and could engage in daily life physical activities. He was rechecked at a local hospital by cardiac ultrasound, where his left ventricular ejection fraction was above 50%.

## 3. Discussion

This case report describes a critically patient with myocardial infarction complicated by CS due to complete occlusion at the origin of the LMCA. Despite limited medical resources, significant therapeutic efficacy was achieved performing only DCB angioplasty. Following failed thrombolysis therapy, the treatment team performed only DCB without thrombus aspiration, stent implantation, or MCS. The door-to-balloon time was extremely short, with the entire procedure completed in just 15 minutes. Postoperatively, the patient’s shock was reversed. One month later, follow-up at a tertiary hospital confirmed via IVUS that no further intervention was required for the LMCA. Six months post-procedure, cardiac function had recovered well.

Early and correct diagnosis is a prerequisite for effective treatment, so it is important to emphasize the diagnostic and assessment value of ECG for the patient’s condition. The ECG of LMCA-AMI often shows ST-segment depression ≥ 0.1 mV in 6 or more leads and ST-segment elevation in lead aVR and/or V1, which may also be combined with changes such as leftward deviation of the electrical axis, prolongation of the QTc interval, and QRS widening.^[3]^

We must first candidly acknowledge that the strategy adopted in this case represents a “reluctant deviation” from current international guidelines. The European Society of Cardiology and American College of Cardiology/American Heart Association guidelines unequivocally stipulate that LMCA lesions require coronary artery bypass grafting or PCI with drug-eluting stent implantation, with intravascular imaging guidance such as IVUS being the recommended method (Class IIa) for optimizing the efficacy of interventional therapy and reducing events.^[4,5]^ Concurrently, CS warrants active consideration of circulatory support via IABP or ECMO.^[6]^ However, this case presented unique circumstances: during the pandemic, our hospital lacked access to IVUS and IABP, and our institution did not yet perform ECMO. Faced with a patient exhibiting extremely high mortality during transfer, the treatment team opted neither for passive waiting nor risky referral. Instead, based on available resources, we made the boldest and most rational decision possible.

The key to success lies in returning to the “first principle” of reperfusion therapy: restoring blood flow as rapidly as possible. Preoperative CAG revealed complete occlusion of the left main trunk from its origin, yet only 30% residual stenosis remained after DCB angioplasty. This striking radiological discrepancy suggests that the culprit of this acute event was not severe calcified or fibrotic plaque, but rather a massive fresh thrombus formed on the basis of mild to moderate plaque. Although the rhTNK-tPA thrombolysis performed at the referring hospital was assessed as “no recanalization,” it likely significantly degraded the thrombus’s fibrin scaffold, transforming it from a refractory occlusion to a “compressible/fragmentable” state. This may have laid the crucial foundation for the DCB to successfully compress and fragment the thrombus through pure mechanical expansion force, thereby restoring blood flow. Conversely, implanting a stent in this thrombus-rich lesion would significantly increase the risk of early and late in-stent thrombosis due to stent encapsulation. Moreover, forced stent implantation without IVUS assessment of apposition and sizing carries extremely high risks of incomplete expansion, poor apposition, and long-term thrombosis.

The decision to forego thrombus aspiration was primarily driven by an acute onset of left heart failure intra-procedure, making rapid revascularization imperative. Given the immediate restoration of TIMI-3 flow with balloon dilation and the subsequent resolution of shock, pursuing aspiration was deemed an unnecessary delay. Furthermore, a dedicated catheter was not available during the pandemic.

For CS, any additional procedures performed in pursuit of “perfection” or “guideline compliance” – such as waiting for devices or performing complex maneuvers – consume the patient’s chances of survival. Rapid restoration of coronary blood flow is itself the most effective circulatory support. DCB technology achieves “intervention without stenting,” not only providing immediate revascularization but also delivering the antiproliferative drug paclitaxel to prevent long-term restenosis.^[7]^ Restoring LMCA flow immediately breaks the vicious cycle of shock, yielding superior outcomes compared to any subsequently implanted mechanical support device. It avoids risks associated with metallic implants, preserves future treatment options for patients, and enables the ideal outcome of “no intervention required” confirmed by IVUS after one month.

Although successful in this specific context, the procedure was performed without IVUS – a cornerstone of guideline-recommended, optimized left main PCI for lesion assessment and stent optimization. The lack of IVUS underscores that this DCB-only strategy was a necessary contingency under constrained conditions, rather than a replacement for standard imaging-guided therapy. IVUS- and OCT-guided stent implantation is associated with better long-term results compared to angiography alone.^[8]^

## 4. Conclusion

This case demonstrates that in resource-limited settings, when safe stent implantation cannot be guaranteed for thrombotic left main occlusion, the DCB-assisted rapid reperfusion strategy may represent a viable, life-saving option. It offers a new approach for primary care hospitals managing such critical emergencies.

## Acknowledgments

We thank the patient for allowing the publication of this case report. Not applicable.

## Author contributions

**Conceptualization:** Di Wang.

**Investigation:** Taiyun Chu, Yuanyuan Zhou, Yudi Wang.

**Supervision:** Shihong Luo.

**Writing – original draft:** Guochun Hu.

## References

[1] Chinese Society of Cardiology. Guidelines for the diagnosis and treatment of acute ST-segment elevation myocardial infarction (2019). Chin J Cardiol. 2019;47:766–83. 10.3760/cma.j.issn.0253-3758.2019.10.003 31648459

[2] Byrne RA Rossello X Coughlan JJ; ESC Scientific Document Group. 2023 ESC guidelines for the management of acute coronary syndromes. Eur Heart J. 2023;44:3720–826. 37622654 10.1093/eurheartj/ehad191

[3] D’Angelo C Zagnoni S Gallo P Tortorici G Casella G Di Pasquale G. Electrocardiographic changes in patients with acute myocardial infarction caused by left main trunk occlusion. J Cardiovasc Med (Hagerstown). 2018;19:439–45. 29889168 10.2459/JCM.0000000000000684

[4] Sousa-Uva M Neumann FJ Ahlsson A; ESC Scientific Document Group. 2018 ESC/EACTS guidelines on myocardial revascularization. Eur J Cardiothorac Surg. 2019;55:4–90. 30165632 10.1093/ejcts/ezy289

[5] Lawton JS Tamis-Holland JE Bangalore S. 2021 ACC/AHA/SCAI guideline for coronary artery revascularization. J Am Coll Cardiol. 2022;145:e771. 10.1016/j.jacc.2021.09.006 34895950

[6] van Diepen S Katz JN Albert NM; American Heart Association Council on Clinical Cardiology; Council on Cardiovascular and Stroke Nursing; Council on Quality of Care and Outcomes Research; and Mission: Lifeline. Contemporary management of cardiogenic shock: a scientific statement from the American Heart Association. Circulation. 2017;136:e232–68. 28923988 10.1161/CIR.0000000000000525

[7] Jeger RV Eccleshall S Wan Ahmad WA; International DCB Consensus Group. Drug-coated balloons for coronary artery disease: third report of the International DCB Consensus Group. JACC Cardiovasc Interv. 2020;13:1391–402. 32473887 10.1016/j.jcin.2020.02.043

[8] Şaylik F Hayiroglu MI Akbulut T Çinar T. Comparison of long-term outcomes between intravascular ultrasound-, optical coherence tomography- and angiography-guided stent implantation: a meta-analysis. Angiology. 2024;75:809–19. 37644871 10.1177/00033197231198674

